# Adverse childhood experiences screening in pediatric primary care and changes in the rate of visits to social work and behavioral health

**DOI:** 10.1186/s12887-025-05456-4

**Published:** 2025-02-08

**Authors:** Sonya Negriff, Margo Sidell, Lee Barton, Mercie J. DiGangi

**Affiliations:** 1 Department of Research & Evaluation, Kaiser Permanente Southern California, 100 S Los Robles Ave, Pasadena, CA 91101 USA; 2 Department of Pediatrics, Kaiser Permanente Southern California, Pasadena, CA USA; 3 Kaiser Permanente Bernard J Tyson School of Medicine, Pasadena, CA 91101 USA

**Keywords:** Adverse childhood experiences, Screening, Pediatrics, Behavioral health, Referral

## Abstract

**Background:**

There is increasing interest in screening for adverse childhood experiences in pediatric primary care, but no evidence of the actual consequences on behavioral/mental health services. This study tested the association between initiation of ACEs screening in pediatric primary care and changes in the rate of referrals to social work and visits to social work and behavioral health.

**Methods:**

Data came from the electronic health records of children and adolescents between 2 and 18 years old who were members of a large integrated healthcare system serving Southern California (*N* = 513,812). Poisson regression was used to compare the rate of referrals and visits to social work and behavioral health visits for clinics doing standardized ACEs screening (i.e., intervention clinics; *n* = 28) versus clinics not screening (i.e., control clinics; *n* = 64) during June 1-December 31 2022 as well as for these same months in 2020 and 2021.

**Results:**

Intervention clinics had an average screening rate of 57% (range 26.8 to 91.9%) and an average positive screen rate of 11% (range 1.6–25.1%). The difference in the adjusted rate from 2021 to 2022 was significantly different between intervention and control clinics for referrals to social work (RR 1.48, 95% CI 1.25, 1.74), but was not statistically different for visits to social work or behavioral health.

**Conclusions:**

The findings suggest that ACEs screening does not significantly increase the rates of social work and behavioral health visits, although it did increase referrals to social work. We acknowledge that this may vary based on geographic areas and populations served by different healthcare systems.

## Introduction

Adverse childhood experiences (ACEs) include child abuse, neglect, and household dysfunction (i.e., parental mental illness, incarceration, substance use, intimate partner violence, divorce) that occurred before the age of 18 [1]. Surveillance data show that experiencing ACEs is highly prevalent in the U.S., with 44.3% of children experiencing one or more ACE [2]. In turn, there is consistent evidence that ACEs increase the risk for a variety of physical and mental health problems at the population level [3, 4]. In order to combat the negative sequalae of ACEs, the American Academy of Pediatrics recommended over a decade ago to incorporate discussions regarding ACEs in pediatric primary care visits, although not ACEs screening specifically, and emphasized the importance of trauma-informed care [5]. In 2020, California was the first state in the US to reimburse for standardized ACEs screening once a year for all children with Medicaid/MediCal coverage [6]. In addition, recommendations for clinical cut offs and referrals for positive screening were published, with a referral to a mental health provider as the primary action for a positive ACEs screening [6]. However, this approach raises several concerns. First is the issue of whether the suggested cut-offs are sufficient for adequately individualizing referrals to mental health. As the impact of the types of ACEs varies widely and other protective factors may mitigate ACEs and prevent symptoms, a cut-off may not appropriately identify those in need of treatment. Second, because the primary treatment for ACEs is a referral to mental health, it is possible that initiation of ACEs screening could overwhelm already overburdened mental health providers. While simulations have indicated the possibility that the demand for services may far outweigh the availability, this depends on a number of parameters that are unknown, including the number of patients who complete screenings, the rate of positive ACEs screenings in the population, the number of mental health providers, and the uptake of services [7]. As of yet, there has been no real-world data to indicate whether ACEs screening may increase the need for services from mental health providers and if children are receiving the services they need.

A previous study within Kaiser Permanente Southern California tested the change from a pilot ACEs screening and referral process in which pediatricians directly referred positive screens to behavioral health (i.e., mental health), to an updated process that included using a social worker for triage and referral to behavioral health [8]. That study found that the change in the ACEs screening and referral process, likely tied to the inclusion of the social worker, significantly increased the rate of completed visits to behavioral health (receipt of services) and therefore this process became the standard for all clinics. However, the study was not able to address whether the *initiation* of standardized ACEs screening and referral is associated with increased demand for either social work or behavioral health services. With the impetus for routine ACEs screening growing, it is necessary to provide evidence demonstrating the impact on social worker and behavioral health providers in order to strengthen the knowledge base for ACEs screening initiatives.

### The current study

To fill this need for evidence of the consequences of ACEs screening on social work and behavioral health services, the present study examined the rate of referrals to social work, visits to social work, and visits to behavioral health after initiation of ACE screening within a large integrated healthcare delivery system. We first compared rates of referral and visits to social work and visits to behavioral health from June through December 2022 after ACEs screening was implemented at 28 clinics (i.e., intervention clinics) to the same period one and two years prior to determine if there was an increase in referrals and visits within these same clinics. However, we were concerned that increases in 2022 within the intervention clinics may reflect COVID-19 pandemic-related increases in mental health service needs rather than only the effects of ACEs screening. To address this, we also compared the rates of referrals and visits at the intervention clinics to the remaining 64 clinics that did not initiate standardized ACEs screening (i.e., control clinics). These comparisons for intervention versus control clinics were tested for 2022, as well as for the two years prior. In doing so we were able to determine whether the rates were significantly different for intervention versus control clinics within each year—differences in 2022 but not in 2021 and 2020 would indicate an effect of ACEs screening. Additionally, we were able to test whether the time trends were changing similarly from 2020 to 2022 for both intervention and control clinics— differences in the rates from 2020/2021 to 2022 only for the intervention clinics would indicate an effect of ACEs screening. This approach allowed for the most rigorous test by employing both a within-clinic repeated measures, as well as intervention versus control repeated measures design.

## Methods

### Setting and study population

The study was conducted at Kaiser Permanente Southern California (KPSC), an integrated healthcare delivery system serving more than 4.6 million members, including approximately 1 million children. The study was reported in accordance with the Strengthening Reporting of Observational Studies in Epidemiology (STROBE) reporting guidelines [9].

The data for the current study were obtained from the electronic health record (EHR) of children and adolescents between ages 2 and 18 years old who were members of the healthcare system and had an eligible healthcare visit between June 1 and December 31, 2020, 2021 or 2022. An eligible visit was determined as a pediatric primary care visit that would be flagged for ACEs screening (i.e., any non-urgent pediatric office visit).

### Design

To assess whether the initiation of ACEs screening was associated with an increase in the rate of referrals to social work, visits to social work, and visits to behavioral health we compared the same seven-month time period (June 1-December 31) in 2020, 2021 and 2022 for the intervention and control clinics as a repeated measures design.

### Intervention

ACEs screening was implemented June 1st 2022 at 28 pediatric clinics across the KPSC service area. At least one clinic within each Medical Center Area was chosen to participate. The Child Abuse Champion from each Medical Center Area selected at least one clinic that was ready to implement the ACEs screening. The ACEs screener, Pediatric ACEs and Related Life-events Screener (PEARLS) [10] included the 10 ACEs questions from the original KP-Centers for Disease Control and Prevention ACEs questionnaire [1] plus seven additional questions assessing neighborhood/community violence exposure, discrimination, housing instability, food insecurity, separation from parent due to foster care or immigration, parent/guardian death, and parental serious physical illness or disability. The score on part 1 was used for MediCal reimbursement but the score on both parts was used to determine a positive screen. The criteria for a positive screen were: (1) score of 1 or more ACEs AND (2) behavioral and/or mental health symptoms (e.g., developmental delays, learning difficulties, falling grades, attention problems, anxiety, depression, parent/child relationship problems, discipline problems at school or home). These symptoms are routinely assessed during pediatric visits. The pediatrician referred any positive screen) to a medical social worker, the social worker completed a psychosocial assessment to identify service needs including behavioral health, parenting classes, food insecurity, and housing insecurity. If behavioral health services were indicated, the social worker provided a warm handoff by directly connecting the family to behavioral health via phone. This referral process has been shown to facilitate receipt of behavioral health services [8]. In this process the social worker served as a triage point rather than treatment provider. While referral to other services such as food banks and parenting classes were made, because these are external to our healthcare system it is very difficult to track these referrals. As such the current study only tested social work and behavioral health referrals and visits. Anticipatory counseling regarding the harms of ACEs was done universally to destigmatize these experiences. When done in a non-judgmental and empathetic way, providers can destigmatize these experiences and educate patients on the connection to mental and physical health. For this study, pre-intervention years were 2020 and 2021 while post-intervention was 2022.

### Control

The remaining 64 clinics that did not initiate the standardized ACEs screening were used as the control group.

### Outcomes

There were three outcomes (1) the rate of referrals to social work, (2) the rate of completed visits to social work, and (3) the rate of completed visits to behavioral health. The denominator for all rates was the eligible pediatric population between June 1 and December 31st for three time points; 2020, 2021, and 2022. All visit data were obtained from the EHR.

### Covariates

Several covariates from the EHR were included in the analyses based on the literature in this area with regards ACEs prevalence [11, 12] and to adjust for differences between pragmatically-selected intervention and control clinics. These included age at screening (2–5 years, 6–10 years, 11–18 years), sex (male, female), race/ethnicity (Non-Hispanic White, Non-Hispanic Black, Hispanic, Asian/Pacific Islander, Other/Unknown), Medicaid/MediCal status (yes/no), and Medical Center.

### Ethics statement

The evaluation of the ACEs clinical initiative was determined as exempt by the Kaiser Permanente Southern California Institutional Review Board with a waiver of informed consent.

### Statistical analyses

Differences in demographic characteristics within years between intervention and control clinics were calculated using the chi-squared test. Poisson models were calculated using generalized estimating equations to account for multiple records per person to estimate the association between each of the three outcomes (i.e., referrals to social work, completed visits to social work and completed visits to behavioral health), intervention category (intervention or control) and three time points (2020, 2021, and 2022) in this repeated measures framework. Poisson regression is a common model when assessing rates. This was performed using SAS proc genmod. The unadjusted model included a binary indicator for category, a time indicator for the three time points, and an interaction term between the two. This model also included individual in the repeated statement. This design tested whether (a) the rate of completed visits was different between 2020, 2021, and 2022 within each group, (b) there was a difference in rates of referrals or completed visits between intervention and control clinics at each time point, and (c) the change between 2020, 2021, and 2022 was different between intervention and control clinics. Models were further adjusted for age, sex, race/ethnicity, MediCal status, and medical center. When calculating the time point effect comparisons, a Bonferroni correction was implemented for multiple comparisons and 2 sided *p* <.0125 was used to indicate statistical significance. Results are presented as relative risk and 95% confidence intervals as well as estimated rates of referrals to social work and completed visits to social work or behavioral health calculated from the unadjusted and fully adjusted models using proc plm and estimate statements with appropriate coding for group, time, and the group by time interaction. All analyses were performed using SAS version 9.4 [13]. We excluded 508 individuals with missing sex as we could not accurately assess the effect of sex when they were included in the model. There was no missingness on any of the other covariates.

### Role of the funding source

The funding source had no involvement in the design, collection, analyses, interpretation of the data, writing the report, or decision to submit for publication.

## Results

### Descriptives

The analysis cohort consisted of *N* = 513,304 distinct participants (excluding 508 individuals with missing sex). Intervention clinics had an average screening rate of 57% (range 26.8 to 91.9%) and an average positive screen rate of 11% (range 1.6–25.1%). Table 1 shows the demographic composition of the cohort at each of the three time periods. Across years, the majority of participants were in the oldest age category [11–18] (41.6%, 41.2%, and 40% for intervention clinics in years 2020, 2021, and 2022, respectively), followed by age 2–5 in 2020 (32.5% for intervention clinics), and 6–10 (25.9% in intervention clinics in year 2020). More children were in the 6–10 age group than 2–5 in years 2021 and 2022 (31.2% and 27.5% for intervention clinics in 2021 for age 6–10 and 2–5, respectively and 31.3% and 28.7% for intervention clinics in 2022 for age 6–10 and 2–5, respectively). The population was similar between females and males at each time point (range from 48.5 to 50.2% female across years and group). People of Hispanic race/ethnicity made up most of the cohort in all three years (56.7% for intervention clinics in all 3 years) and this was higher in intervention clinics compared to controls (56.7% for intervention clinics and 48.1% for control clinics in 2020) (Table 1). Control clinics had higher white and Asian populations each year, while intervention clinics had a higher number of participants identified as Black (9.3% vs. 5.4% for intervention and control clinics in 2020). The percent of each cohort with MediCal ranged from 21.8% in 2020 to 22.9% in 2022 for the control group and 26.4% in 2020 to 28.3% in 2022 in the intervention group. Unadjusted and adjusted estimate rates for the three outcomes for each year and clinic group can be found in Table 2.

**Table 1:** Cohort description at 3 time points for *N* = 513,304 individuals^a*^

	2020			2021			2022		
	Control	Intervention	p-value	Control	Intervention	p-value	Control	Intervention	p-value
	(*N* = 148234)	(*N* = 84343)		(*N* = 168325)	(*N* = 95313)		(*N* = 159928)	(*N* = 87555)	
**Age Category (years)**			< 0.0001			0.004			< 0.0001
Age 11–18	62,237 (42.0)	35,078 (41.6)		70,248 (41.7)	39,311 (41.2)		67,165 (42.0)	35,010 (40.0)	
Age 2–5	46,796 (31.6)	27,433 (32.5)		45,408 (27.0)	26,253 (27.5)		44,043 (27.5)	25,169 (28.7)	
Age 6–10	39,201 (26.4)	21,832 (25.9)		52,669 (31.3)	29,749 (31.2)		48,720 (30.5)	27,376 (31.3)	
**Sex**			< 0.0001			0.002			< 0.0001
Female	72,427 (48.9)	42,345 (50.2)		81,671 (48.5)	46,846 (49.1)		77,676 (48.6)	43,336 (49.5)	
Male	75,807 (51.1)	41,998 (49.8)		86,654 (51.5)	48,467 (50.9)		82,252 (51.4)	44,219 (50.5)	
**Race/Ethnicity**			< 0.0001			< 0.0001			< 0.0001
Asian/Pacific Islander	19,387 (13.1)	6650 (7.9)		24,068 (14.3)	7769 (8.2)		23,148 (14.5)	7287 (8.3)	
Black	7993 (5.4)	7876 (9.3)		9944 (5.9)	8826 (9.3)		9227 (5.8)	7813 (8.9)	
Hispanic	71,314 (48.1)	47,817 (56.7)		79,248 (47.1)	54,046 (56.7)		74,495 (46.6)	49,666 (56.7)	
Other	11,526 (7.8)	5498 (6.5)		14,264 (8.5)	6768 (7.1)		14,350 (9.0)	6800 (7.8)	
White	38,014 (25.6)	16,502 (19.6)		40,801 (24.2)	17,904 (18.8)		38,708 (24.2)	15,989 (18.3)	
**MediCal**			< 0.0001			< 0.0001			< 0.0001
No	115,965 (78.2)	62,095 (73.6)		130,653 (77.6)	69,023 (72.4)		123,343 (77.1)	62,741 (71.7)	

Note: ^a^person can be included at multiple time points and can be in intervention and control at different timepoints

^*^Difference assessed within years using the Chi-squared test

**Table 2:** Unadjusted and adjusted estimated rates in percent (95% confidence interval) of three outcomes by year and clinic group

		Unadjusted^a^		Adjusted	
Outcome	Year	Intervention	Control	Intervention	Control
Referral to Social Work	2020	0.36 (0.32,0.41)	0.34 (0.31,0.37)	0.31 (0.27,0.35)	0.20 (0.18,0.22)
	2021	0.44 (0.40,0.48)	0.39 (0.37,0.43)	0.40 (0.36,0.44)	0.23 (0.21,0.25)
	2022	0.66 (0.61,0.71)	0.40 (0.37,0.43)	0.60 (0.54,0.66)	0.24 (0.21,0.26)
Visit to Social Work	2020	0.43 (0.39,0.48)	0.55 (0.52,0.59)	0.41 (0.37,0.47)	0.34 (0.31,0.37)
	2021	0.48 (0.44,0.53)	0.48 (0.44,0.51)	0.49 (0.44,0.54)	0.29 (0.27,0.32)
	2022	0.60 (0.55,0.65)	0.50 (0.47,0.54)	0.60 (0.55,0.66)	0.31 (0.29,0.34)
Visit to Behavioral Health	2020	5.70 (5.54,5.86)	5.23 (5.11,5.34)	4.58 (4.44,4.73)	4.28 (4.17,4.40)
	2021	6.76 (6.60,6.92)	6.42 (6.30,6.53)	5.43 (5.27,5.59)	5.21 (5.08,5.33)
	2022	7.31 (7.14,7.49)	6.80 (6.68,6.92)	5.92 (5.75,6.09)	5.53 (5.39,5.66)

Note: ^a^Models adjusted for age, sex, race/ethnicity, MediCal status, and medical center

### Rates of referrals and completed visits over time: within clinic group effects

In fully adjusted models testing the effect of ACEs screening on referrals to social work for 2020, 2021, and 2022, participants in both the intervention and control groups were more likely to have a referral to social work in 2022 than in 2020 (RR 1.91 95% CI 1.67, 2.20, RR 1.17 95% CI 1.04, 1.31 respectively). Participants in the intervention group were also more likely to have a referral to social work in 2021 compared to 2020 and in 2022 compared to 2021 (RR 1.27 95% CI 1.10, 1.47, RR 1.50 95% CI 1.33, 1.70 respectively) (Table 3).

**Table 3:** Unadjusted and adjusted relative risk (RR) and 95% confidence interval for the association of time and three outcomes within clinic group

		Unadjusted		Adjusted^a^	
Outcome	Year	Intervention	Control	Intervention	Control
Referral to Social Work	2022 vs. 2021	1.49 (1.32,1.69) ^*^	1.02 (0.91,1.13)	1.50 (1.33,1.70) ^*^	1.02 (0.92,1.13)
	2021 vs. 2020	1.21 (1.05,1.40) ^*^	1.16 (1.03,1.30)	1.27 (1.10,1.47) ^*^	1.15 (1.02,1.28)
	2022 vs. 2020	1.81 (1.58,2.08) ^*^	1.18 (1.05,1.32) ^*^	1.91 (1.67,2.20) ^*^	1.17 (1.04,1.31) ^*^
Visit to Social Work	2022 vs. 2021	1.24 (1.09,1.40) ^*^	1.06 (0.96,1.16)	1.24 (1.10,1.40) ^*^	1.07 (0.97,1.17)
	2021 vs. 2020	1.11 (0.97,1.27)	0.86 (0.78,0.95) ^*^	1.17 (1.02,1.34)	0.86 (0.78,0.94) ^*^
	2022 vs. 2020	1.38 (1.20,1.57) ^*^	0.91 (0.83,1.00)	1.45 (1.27,1.66) ^*^	0.92 (0.83,1.01)
Visit to Behavioral Health	2022 vs. 2021	1.08 (1.05,1.12) ^*^	1.06 (1.03,1.09) ^*^	1.09 (1.06,1.12) ^*^	1.06 (1.04,1.09) ^*^
	2021 vs. 2020	1.19 (1.15,1.23) ^*^	1.23 (1.20,1.26) ^*^	1.18 (1.15,1.23) ^*^	1.22 (1.18,1.25) ^*^
	2022 vs. 2020	1.28 (1.24,1.33) ^*^	1.30 (1.27,1.34) ^*^	1.29 (1.25,1.34) ^*^	1.29 (1.26,1.33) ^*^

Note: ^a^Models adjusted for age, sex, race/ethnicity, MediCal status, and medical center; *p <.0125 refers to significance between time contrast (e.g., 2021 vs. 2020) within clinic group

For visits to social work, fully adjusted models indicated that people in the intervention group were more likely to have a visit in 2022 compared to 2021 (RR 1.24 95% CI 1.10, 1.40) and in 2022 compared to 2020 (RR 1.45 95% CI 1.27, 1.66) (Table 3). This was not the case for those in the control group, who were less likely to visit social work in 2021 compared to 2020 (RR 0.86 95% CI 0.78, 0.94) (Table 3).

The fully adjusted models testing the effect of ACEs screening on behavioral health visits across time, within intervention and control clinics separately, (Table 3) showed that participants were more likely to visit behavioral health in 2022 than in 2020 and 2021 in both intervention and control clinics (2020 RR 1.29 95% CI 1.25, 1.34, RR 1.29 95% CI 1.26, 1.33 respectively; 2021 RR 1.18, 95% CI 1.15, 1.23, RR 1.22 95% CI 1.18, 1.25, respectively).

### Rates of referrals and completed visits within each year: between clinic group effects

In fully adjusted models, participants in the intervention clinics were more likely than those in the controls clinics to have a referral to social work in all 3 years (2020 RR 1.55 95% CI 1.34, 1.79, 2021 RR 1.72 95% CI 1.51, 1.95, 2022 RR 2.54 95% CI 2.26, 2.85) (Table 4).

**Table 4:** Unadjusted and adjusted* relative risk (RR) and 95% confidence interval for the association of three outcomes at three time points for intervention vs. control clinics

		Unadjusted	Adjusted^a^
Outcome	Year	Intervention vs. Control	Intervention vs. Control
Referral to Social Work	2020	1.06 (0.92,1.23)	1.55 (1.34,1.79)*
	2021	1.11 (0.99,1.26)	1.72 (1.51,1.95)*
	2022	1.64 (1.46,1.83)*	2.54 (2.26,2.85)*
Visit to Social Work	2020	0.78 (0.69,0.89)*	1.21 (1.07,1.37)*
	2021	1.01 (0.90,1.13)	1.65 (1.46,1.86)*
	2022	1.18 (1.06,1.32)*	1.92 (1.70,2.16)*
Visit to Behavioral Health	2020	1.09 (1.05,1.13)*	1.07 (1.03,1.11)*
	2021	1.05 (1.02,1.09)*	1.04 (1.01,1.08)
	2022	1.08 (1.04,1.11)*	1.07 (1.03,1.11)*

Note: ^a^Models adjusted for age, sex, race/ethnicity, MediCal status, and medical center; *p <.0125 (Bonferroni correction)

Participants were also more likely in intervention clinics than control clinics to visit social work at all three time points (2020 RR 1.21 95% CI 1.07, 1.37, 2021 RR 1.65 95% CI 1.46, 1.86, 2022 RR 1.92 95% CI 1.70, 2.16) (Table 4).

Models comparing the rates of behavioral health visits for the intervention versus control clinics within each year (2020, 2021, 2022) showed that the adjusted rates were significantly higher in the intervention clinics than those in the control clinics in 2020 and 2022 (RR 1.07 95% CI 1.03, 1.11, RR 1.07, 95% CI 1.03, 1.11 respectively; Table 4).

### Rate of referrals and completed visits over time: between clinic group effects

Fully adjusted models to determine whether the increase over time in social work referrals was different for the intervention clinics and the control clinics showed that the increase from 2021 to 2022 was higher for the intervention clinics compared to control (RR 1.48, 95% CI 1.25, 1.74) (Table 5). The increase from 2020 to 2021 was not statistically different between groups.

**Table 5:** Unadjusted and adjusted* relative risk (RR) and 95% confidence interval for the association of intervention vs. control and three outcomes over time

		Unadjusted		Adjusted^a^	
Outcome	Contrast	Intervention VS Control	*p*	Intervention VS Control	*p*
Referral to Social Work	2022 vs. 2021 Intervention VS 2022 vs. 2021 Control	1.47 (1.25,1.73)	< 0.0001	1.48 (1.25,1.74)	< 0.0001
	2021 vs. 2020 Intervention VS 2021 vs. 2020 Control	1.05 (0.87,1.26)	0.6166	1.11 (0.92,1.34)	0.266
Visit to Social Work	2022 vs. 2021 Intervention VS 2022 vs. 2021 Control	1.17 (1.00,1.37)	0.0482	1.16 (0.99,1.36)	0.0598
	2021 vs. 2020 Intervention VS 2021 vs. 2020 Control	1.29 (1.09,1.52)	0.003	1.36 (1.15,1.61)	0.0003
Visit to Behavioral Health	2022 vs. 2021 Intervention VS 2022 vs. 2021 Control	1.02 (0.98,1.06)	0.3116	1.03 (0.99,1.07)	0.1803
	2021 vs. 2020 Intervention VS 2021 vs. 2020 Control	0.97 (0.93,1.01)	0.1226	0.98 (0.93,1.02)	0.25

Note: ^a^Models adjusted for age, sex, race/ethnicity, MediCal status, and medical center

For visits to social work, the difference was significantly different from 2020 to 2021, with intervention clinics having a higher increase (RR 1.36, 95% CI 1.15, 1.61) (Table 5). The increase from 2021 to 2022 was not significantly different between groups.

Models testing the rate of change over time from 2020 to 2021 and 2021 to 2022 found no significant difference between groups for behavioral health visits, (RR 1.03, 95% CI 0.99, 1.07, RR 0.98, 95% CI 0.93, 1.02 respectively; Table 5), indicating that behavioral health visits were increasing at similar rates for both the intervention and control clinics and not likely due to initiation of ACEs screening.

## Discussion

This study provides the first evidence linking the initiation of ACEs screening in pediatric primary care with changes in service volumes for behavioral health providers. Our findings show that after ACEs screening was implemented there was a significant difference between intervention and control clinics in the rate of referrals to social work from 2021 to 2022. However, there were no significant differences between the intervention and control clinics in the rate of completed visits to social work or behavioral health. Although there were higher rates of behavioral health visits in intervention clinics in both 2021 and 2022 after covariate adjustment, and significant increases in rates from 2020 to 2022 for both groups, the results did not indicate that this was due to the initiation of ACEs screening. Importantly, although a significant effect, it should be noted that the increase over time was very small, a 1.34% increase from 2020 to 2022 for the intervention clinics and a 1.25% increase from 2020 to 2022 for the control clinics (difference in rates shown in Table 2). Overall, these results indicate that mental health providers may expect a small increase in the rate of visits following the initiation of ACEs screening in an affiliated pediatric primary care, but this increase is not statistically significant.

The application of ACEs scores to healthcare has spurred substantial debate. While some argue that there is little evidence of the clinical utility of particular ACE scores and without evidence-based interventions that link directly to specific ACE scores there will be minimal benefit of screening [14, 15]. Advocates for ACEs screening point to the importance of the screening for prompting conversations with parents about how early experiences can affect the health of their child and an avenue for gaining better information about their needs [16, 17]. Although the current study does not address these issues, it does provide critical data regarding other concerns related to the capacity of behavioral health services after ACEs screening. Importantly, the findings show that initiation of ACEs screening was not associated with a statistically significant increase in rate of visits to social work or behavioral health compared to non-screening clinics. Without such data, perceived increases in service needs may deter healthcare providers from initiating ACEs screening. However, we did find that referrals to social work were higher in 2022 than 2021 for the intervention than control clinics. Coupled with our trend level results regarding visits to social work, this implies that ACEs screening may have increased initial referral to social work, but that due to uncompleted referrals the rate of visits was not statistically higher. The expectation was that visits to behavioral health would increase with the initiation of ACEs screening. This may indicate children with possible behavioral health services needs are not getting treatment. However, it may also indicate the social work triage is working to individualize care, as not all children with a positive screen will need therapy. Some may have housing or food insecurity and those are resources that can be given by a social worker. A critical next step will be to understand if there are family or system level barriers to completing visits with social work or behavioral health. Screening initiatives may need to pay particular attention to this in order to ensure referrals result in completed visits.

A critical component of the referral process in our system was the social worker. In a previous study we tested change from a pilot ACEs screening process to an updated process including the social worker and found that without this additional assessment and referral pathway significantly fewer children who screened positive received behavioral health services [8]. However, in that prior study we did not test going from no screening to a standardized ACEs screening, which is the contribution of the current study. While we recognize that inclusion of a social worker in the screening and referral process may not be feasible for every healthcare provider, without this triage point there may be higher referral rates to behavioral health providers but fewer completed visits.

The results also indicated that there was a similar increase in rates of behavioral health services for both intervention and control clinics from 2020 to 2022. Given the increases in mental health symptoms reported by adolescents as a likely result of the pandemic [18–21], the subsequent increase in utilization of behavioral health services is not surprising. Different conclusions may have been drawn if we had only included the intervention clinics, as we might have assumed there was an increase in 2022 due to the ACEs screening. However, because we included control clinics, we can show that the increase is similar across all our clinics and not due to ACEs screening.

There are several limitations that should be noted. First, our healthcare system is very large and may not be comparable to smaller system or individual providers. In addition, the demographics of our membership population may not reflect the patients serviced in other geographic areas. In terms of demographic differences between the intervention versus control clinics, there were higher percentages of Black and Hispanic children in the intervention clinics. While the adjusted models control for these differences, we understand that there may be inequities in the rates of ACEs as well as engagement with the mental health system that differ by race/ethnicity. Other differences between intervention and control clinics may have been present due to our pragmatic selection of intervention clinics. It is possible these initial clinics (i.e., intervention) had better provider buy-in and resources for supporting screening and referral protocols than non-selected clinics, but we are not able to determine these possible differences as this information is not available in the EHR. As always, more data are needed to complement this study and obtain a more complete understanding of the consequences of ACEs screening. Another consideration of these data is that we did not restrict our sample to those with positive ACEs screening, so the social work and behavioral health visits we captured may have been a combination of children referred for ACEs screening as well as for other reasons. However, because we wanted to provide evidence of changes related to the initiation of ACEs screening, it was necessary to use the entire population in order to compare with the prior years when ACEs screening was not yet implemented. In addition, because screening rates were not 100% at any clinic in the intervention group these data include a small portion of children who were not screened. This is a realistic reflection of actual screening programs as no clinic is expected to have perfect screening rates. As such these data represent likely changes in the behavior health needs of the overall pediatric population when ACEs screening is implemented in a clinic or healthcare system. However, should screening rates be higher within other healthcare systems it is possible that behavioral health need may be higher, highlighting the need to augment these data with those from other clinics or healthcare systems.

## Conclusion

As ACEs screening continues to gain momentum evidence is needed to guide implementation and inform clinical stakeholders that may have concerns with the impact on their services. The findings show no statistically significant increase in service receipt after initiation of ACEs screening, although still a small raw increase, which is aligned with the United States Preventive Services Task Force recommendations that screening should result an increase in need for treatment. We have also shown in previous publications that all children with a social work visit receive a behavioral health or social service referral, indicating ACEs screening is increasing service needs. We acknowledge that the actual rates may differ based on the underlying population being served and further work is needed to round out our understanding of the full consequences of ACEs screening. Importantly, future research including longitudinal follow-up is needed to determine if ACEs screening and associated mental health treatments are effective and lead to population level improvements in ACEs-related health problems.

## Data Availability

The authors do not have permission to share the health care data from this study. The study materials may be requested from the first author.

## Abbreviations

ACEs: Adverse childhood experiences
KPSC: Kaiser Permanente Southern California
HER: Electronic health records

## References

[1] Felitti VJ, Anda RF, Nordenberg D, Williamson DF, Spitz AM, Edwards V, et al. Relationship of childhood abuse and household dysfunction to many of the leading causes of death in adults. The adverse childhood experiences (ACE) study. Am J Prev Med. 1998;14(4):245–58. 9635069 10.1016/s0749-3797(98)00017-8

[2] Walker BH, Brown DC, Walker CS, Stubbs-Richardson M, Oliveros AD, Buttross S. Childhood adversity associated with poorer health: evidence from the US National Survey of Children’s Health. Child Abuse Negl. 2022;134:105871. 36095924 10.1016/j.chiabu.2022.105871

[3] Bright MA, Knapp C, Hinojosa MS, Alford S, Bonner B. The comorbidity of physical, mental, and developmental conditions associated with childhood adversity: a population based study. Matern Child Health J. 2016;20(4):843–53. 26694043 10.1007/s10995-015-1915-7

[4] Baldwin JR, Caspi A, Meehan AJ, Ambler A, Arseneault L, Fisher HL, et al. Population vs individual prediction of poor health from results of adverse childhood experiences screening. JAMA Pediatr. 2021;175(4):385–93. 33492366 10.1001/jamapediatrics.2020.5602 PMC7835926

[5] Garner AS, Shonkoff JP, Siegel BS, Dobbins MI, Earls MF, McGuinn L, et al. Early childhood adversity, toxic stress, and the role of the pediatrician: translating developmental science into lifelong health. Pediatrics. 2012;129(1):e224–31. 22201148 10.1542/peds.2011-2662

[6] ACEs Aware. ACE Screening Clinical Workflows, ACEs and Toxic Stress Risk Assessment Algorithm, and ACE-Associated Health Conditions: For Pediatrics and Adults https://www.acesaware.org/wp-content/uploads/2019/12/ACE-Clinical-Workflows-Algorithms-and-ACE-Associated-Health-Conditions.pdfApril, 2020 [.

[7] Barnett ML, Sheldrick RC, Liu SR, Kia-Keating M, Negriff S. Implications of adverse childhood experiences screening on behavioral health services: a scoping review and systems modeling analysis. Am Psychol. 2021;76(2):364–78. 33734801 10.1037/amp0000756 PMC8161946

[8] Negriff S, DiGangi MJ, Sidell M, Liu J, Coleman KJ. Assessment of screening for adverse childhood experiences and receipt of behavioral health services among children and adolescents. JAMA Netw Open. 2022;5(12):e2247421–e. 36534401 10.1001/jamanetworkopen.2022.47421 PMC9857176

[9] Von Elm E, Altman DG, Egger M, Pocock SJ, Gøtzsche PC, Vandenbroucke JP, Strobe Initiative. The strengthening the reporting of Observational studies in Epidemiology (STROBE) statement: guidelines for reporting observational studies. Ann Intern Med. 2007;147(8):573–7. 17938396 10.7326/0003-4819-147-8-200710160-00010

[10] Koita K, Long D, Hessler D, Benson M, Daley K, Bucci M, et al. Development and implementation of a pediatric adverse childhood experiences (ACEs) and other determinants of health questionnaire in the pediatric medical home: a pilot study. PLoS ONE. 2018;13(12):e0208088. 30540843 10.1371/journal.pone.0208088 PMC6291095

[11] Giano Z, Wheeler DL, Hubach RD. The frequencies and disparities of adverse childhood experiences in the US. BMC Public Health. 2020;20(1):1–12. 32907569 10.1186/s12889-020-09411-z PMC7488299

[12] Crouch E, Probst JC, Radcliff E, Bennett KJ, McKinney SH. Neglect. Prevalence of adverse childhood experiences (ACEs) among US children. Child abuse & neglect. 2019;92:209– 18. 10.1016/j.chiabu.2019.04.010 31003066

[13] SAS version 9.4. Cary, NC, USA: SAS Institute Inc.

[14] Finkelhor D. Screening for adverse childhood experiences (ACEs): cautions and suggestions. Child Abuse Negl. 2018;85:174–9. 28784309 10.1016/j.chiabu.2017.07.016

[15] Anda RF, Porter LE, Brown DW. Inside the adverse childhood experience score: strengths, limitations, and misapplications. Am J Prev Med. 2020;59(2):293–5. 32222260 10.1016/j.amepre.2020.01.009

[16] Watson P. Moving upstream: the case for ACEs screening. Paediatr Child Health. 2019;24(4):274–5. 31241061 10.1093/pch/pxz043 PMC6587400

[17] Gillespie RJ, Folger AT. Feasibility of assessing parental ACEs in pediatric primary care: implications for practice-based implementation. J Child Adolesc Trauma. 2017;10(3):249–56.

[18] Samji H, Wu J, Ladak A, Vossen C, Stewart E, Dove N, et al. Mental health impacts of the COVID-19 pandemic on children and youth–a systematic review. Child Adolesc Mental Health. 2022;27(2):173–89. 10.1111/camh.12501 PMC8653204 34455683

[19] Theberath M, Bauer D, Chen W, Salinas M, Mohabbat AB, Yang J, et al. Effects of COVID-19 pandemic on mental health of children and adolescents: a systematic review of survey studies. SAGE Open Med. 2022;10:20503121221086712. 35371484 10.1177/20503121221086712 PMC8972920

[20] Centers for Disease Control and Prevention. Youth Risk Behavior Surveillance Survey Data Summary & Trends Report: 2011–2021. National Center for HIV, Viral Hepatitis, STD, and TB Prevention; 2021.

[21] Newlove-Delgado T, Russell AE, Mathews F, Cross L, Bryant E, Gudka R, et al. Annual Research Review: the impact of Covid‐19 on psychopathology in children and young people worldwide: systematic review of studies with pre‐and within‐pandemic data. J Child Psychol Psychiatry. 2022. 10.1111/jcpp.13716. 36421049 10.1111/jcpp.13716 PMC10952503

